# The Efficacy and Safety of Acellular Matrix Therapy for Diabetic Foot Ulcers: A Meta-Analysis of Randomized Clinical Trials

**DOI:** 10.1155/2020/6245758

**Published:** 2020-02-01

**Authors:** Wentao Huang, Yongsong Chen, Nasui Wang, Guoshu Yin, Chiju Wei, Wencan Xu

**Affiliations:** ^1^Department of Endocrinology and Metabolism, The First Affiliated Hospital of Shantou University Medical College, 57 Changping Road, Shantou 515041, China; ^2^Shantou University Medical College, 22 Xinling Road, Shantou 515041, China; ^3^Multidisciplinary Research Center, Shantou University, 243 Daxue Road, Shantou 515063, China

## Abstract

**Background:**

Acellular matrix (AM) therapy has shown promise in the treatment of diabetic foot ulcers (DFUs) in several studies. The clinical effects of AM therapy were not well established. Therefore, we conducted a meta-analysis of randomized clinical trials (RCTs) to examine the efficacy and safety of AM therapy for patients with DFUs.

**Methods:**

A literature search of 5 databases was performed to identify RCTs comparing AM therapy to standard therapy (ST) in patients with DFUs. The primary outcome was the complete healing rate and the secondary outcomes mainly included time to complete healing and adverse events.

**Results:**

Nine RCTs involving 897 patients were included. Compared with ST group, patients allocated to AM group had a higher complete healing rate both at 12 weeks (risk ratio (RR) = 1.73, 95% confidence interval (CI): 1.31 to 2.30) and 16 weeks (RR = 1.56, 95% CI: 1.28 to 1.91), a shorter time to complete healing (mean difference (MD) = −2.41; 95% CI: -3.49 to -1.32), and fewer adverse events (RR = 0.64, 95% CI: 0.44 to 0.93).

**Conclusion:**

The present study suggests that AM therapy as an adjuvant treatment could further promote the healing of full-thickness, noninfected, and nonischemia DFUs. AM therapy also has a safety profile. More large well-designed randomized clinical trials with long follow-up duration are needed to further explore the efficacy and safety of AM therapy for DFUs.

## 1. Introduction

Diabetic mellitus, a rapid worldwide epidemic disorder, has become a major global health issue [1]. It is estimated that there are 451 million people with diabetes in 2017, and this number will rise to 693 million people by 2045 [2]. Diabetic foot ulcers (DFUs) are one of the most serious complications of diabetes and account for high levels of morbidity, mortality, and health-care costs [3–5]. The prevalence of DFUs is about 6.3% worldwide, and 19-34% of diabetic patients are liable to suffer from a foot ulceration in their lifetimes [6, 7]. The standard therapy (ST) for DFUs includes debridement, dressing, offloading, vascular assessment, and infection and glycemic control [8]. However, the complete healing rates at 12 and 20 weeks are only 24% and 31%, respectively, for those receiving ST [9]. In addition to ST in DFU care, there are a series of adjuvant therapies being studied, such as acellular matrix (AM) therapy, hyperbaric oxygen therapy, and shockwave therapy [8].

Acellular matrices (AMs) have been used as soft tissue replacement since 1994 [10]. The acellular grafts are processed to remove the cellular components while preserving the three-dimensional structure and the bioactive agents of extracellular matrix, such as collagen, hyaluronic acid, elastin, and fibronectin [11, 12]. Such matrices accelerate ulcer healing by providing structural supports and signals for cellular migration, proliferation, angiogenesis, and endogenous matrix production [13, 14]. Several clinical trials have reported that AM therapy represents a useful adjuvant treatment for DFUs. However, reliable evidences on the clinical effects of AM therapy remain to be addressed. Therefore, the aim of this study was to evaluate the efficacy and safety of AM therapy for DFUs.

## 2. Materials and Methods

The meta-analysis was conducted according to the Preferred Reporting Items for Systematic Reviews and Meta-Analyses (PRISMA) statement [15].

### 2.1. Data Sources and Searches

A comprehensive literature search of PubMed, Embase, the Cochrane Library, and China Biology Medicine disc was performed by two independent researchers (W. H. and Y. C.) to identify RCTs assessing the efficacy and safety of AM in the treatment of DFUs. The last search update was conducted on September 24, 2019. A combination of Medical Subject Terms and keywords to define the concept of diabetic foot and acellular matrix was used, such as “diabetic foot, diabetic ulcer, diabetic wound, foot ulcer, diabetic” and “acellular dermis, acellular matrix, acellular tissue, acellular transplant, acellular graft, decellularized scaffold”. There were no restrictions to language and publication date. In addition, the ClinicalTrial.gov database and reference lists in the selected articles were also searched for any eligible trials and information.

### 2.2. Study Selection

For inclusion in this meta-analysis, literatures needed to meet the following criteria: (1) RCTs consisted of more than 10 patients per group; (2) patients with type 1 or type 2 diabetes suffering from DFUs; (3) controlled trials examining AM therapy versus ST, such as debridement, dressing, offloading, antibiotic treatment, and glycemic control; and (4) studies reporting one of the outcomes at least, including complete healing rate, time to complete heal, ulcer area reduction, ulcer depth reduction, adverse events, and quality of life. The article with the most comprehensive data was included if there were duplicate studies from the same trial. Studies were excluded for the following reasons: (1) reviews, meta-analyses, conference abstracts without available full texts, letters, case reports, trials' protocol, retrospective studies, and animal studies; (2) standard therapy was not the control group or included other experimental treatments, such as growth factor treatment; (3) studies lacking control group; and (4) studies lacking sufficient data of interest. The selection of eligible studies from retrieved articles was independently performed by two investigators (W. H. and N. W.), and disagreements were resolved by consultation with a third investigator (W. X.).

### 2.3. Data Extraction

Two investigators (W. H. and G. Y.) independently extracted the data by using a prepared checklist, and a third investigator (W. X.) was consulted when disagreements arose. The following information was extracted from the eligible studies: the first author's name, year of publication, study design, main inclusion criteria, sample size, population demographics (including age, sex, glycosylated hemoglobin, ankle brachial index, and body mass index), characteristics of the ulcer (grade, area, and duration), information about treatments received, follow-up period, and outcomes. Incidences of the following endpoints were also extracted: completely healed ulcers, time to complete heal, reduction in the ulcer area and depth, adverse events, and quality of life. Complete healing was defined as full epithelialization. To allow an intention-to-treat analysis, the data reflecting the original allocation group were extracted. In addition, data were obtained where possible when they were published on ClinicalTrial.gov database or presented in graphical form in the articles.

### 2.4. Quality Assessment

The risk of bias in the included studies was assessed independently by two investigators (W. H. and C. W.), using the Cochrane Risk of Bias Assessment tool which contained the following domains: random sequence generation, allocation concealment, blinding of participants and personnel, blinding of outcome assessment, incomplete outcome data, selective reporting, and other biases. The risk of bias for each domain was assessed as either unclear, low, or high. Any discrepancies were handled by consultation with the third investigator (W. X.).

### 2.5. Statistical Analysis

All analyses were performed in accordance with the intention-to-treat principles. Differences in continuous outcomes (i.e., time to complete heal) are expressed as mean difference (MD) including 95% confidence interval (95% CI). Differences in dichotomous outcomes (i.e., complete healing rate) are expressed as risk ratio (RR) with 95% CI. Heterogeneity was estimated by the *I*^2^ statistics. At an *I*^2^ ≥ 50%, heterogeneity was considered as significant. A fixed effects model was used in case of low heterogeneity, and a random effects model was used if heterogeneity test revealed statistical significance. Data analyses were performed by Review Manager (RevMan) software (version: 5.3; The Cochrane Collaboration, Copenhagen, Denmark). For outcomes that were reported in ≥5 studies, publication bias was assessed by Begg's test and Egger's test through STATA software (version 16.0; Stata Corp LP, College Station, TX). Sensitivity analysis was performed by deleting each individual study, using the STATA software. All statistical tests were two-sided and a *P* value of <0.05 was considered significant.

## 3. Results

### 3.1. Literature Search

**Figure 1:**
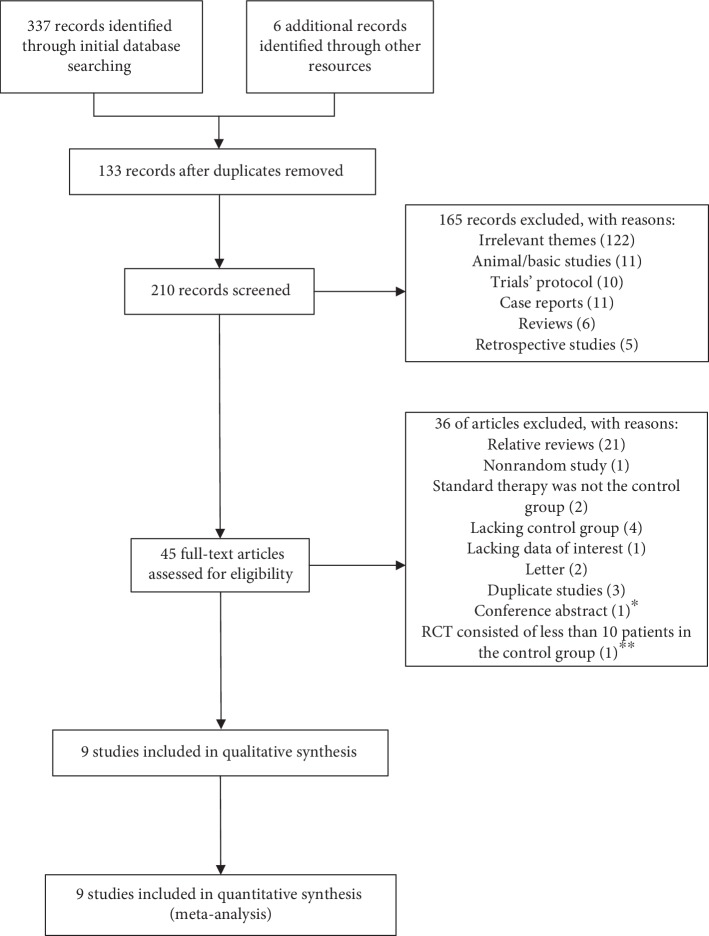
Flow chart of the study selection. ^∗^The article was a conference abstract without available full text and lacked enough information to conduct a quality assessment. ^∗∗^The RCT only included 6 patients in the control group.

A flowchart of the literature screening process is shown in Figure 1. A total of 343 potentially relevant citations were identified, and of which, 133 citations were excluded for duplication. Then, screening of titles and abstracts resulted in the removal of 165 citations in accordance with the inclusion or exclusion criteria. After reading the full texts, 36 articles were excluded, three of them were duplicate studies [16–18], one of them was a conference abstract without available full text [19], while the other one only included 6 patients in the control group [20]. Finally, 9 RCTs were eligible for this meta-analysis [21–29].

### 3.2. Study Characteristics

The characteristics of the included studies are presented in Table 1. A total of 897 patients with DFU were included, 469 receiving AM therapy plus ST and 428 receiving merely ST. The most commonly used ST included debridement, offloading, dressing, and antibiotic treatment. The main entry criteria for the different studies were similar, as shown in Table 1(a). Obese elderly patients accounted for the majority of the recruited population in most studies, with an average age of more than 55 years and an average BMI of more than 28 kg/m^2^. Patients usually had an adequate circulation to the effected extremity. The great majority of the included foot ulcers were full thickness which corresponded to Wagner grades 1 and 2 or University of Texas grades 1-2, noninfective, chronic and refractory, while DFUs in one study [27] were Wagner grade 3. Sample sizes ranged from 14 to 154 patients and follow-up period varied between 4 weeks and 28 weeks.

### 3.3. Quality Assessment

**Figure 2:**
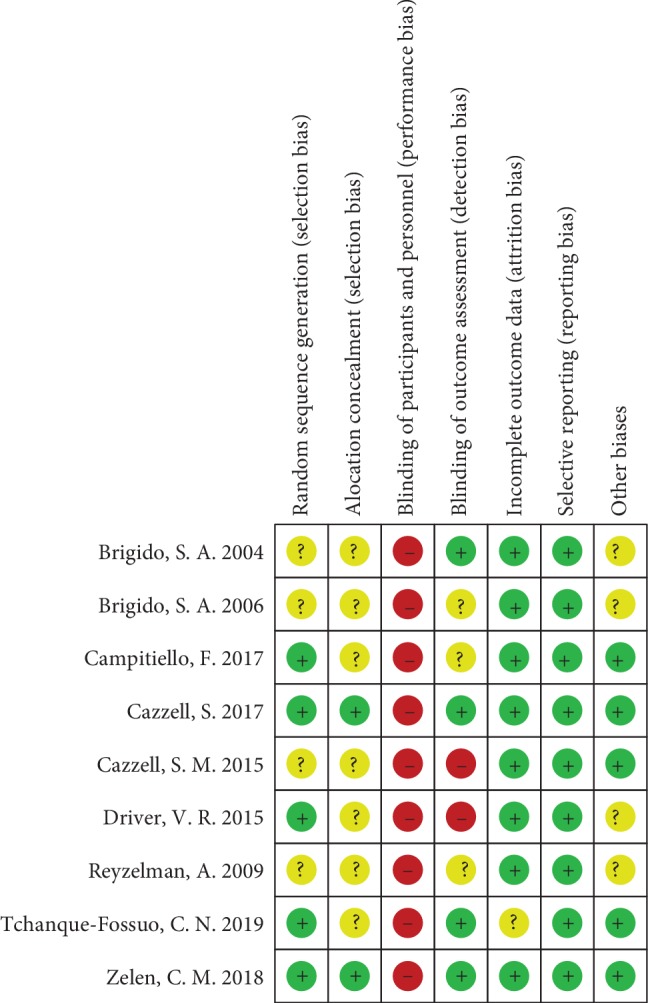
Risk of bias summary.

The results of the bias assessment are presented in Figure 2. All studies were described as RCTs, of which 5 studies [24, 26–29] clearly described the method of random sequence generation and 2 studies [26, 28] reported allocation concealment. Although the blinding method of participants and personnel were not mentioned in 3 studies [22, 23, 27], all 9 studies were assessed as having a high risk of performance bias, as AM was easily to be identified during the application by the study staff. Blinding of outcome assessment was reported in only 4 of the studies [21, 26, 28, 29], and most studies were considered to have a low risk of attrition bias and reporting bias.

### 3.4. Clinical Results

The incidence of complete healed ulcers was the primary outcome. Time to complete heal, ulcer area reduction, ulcer depth reduction, adverse events, and quality of life served as secondary outcomes.

#### 3.4.1. Complete Healing Rate

**Figure 3:**
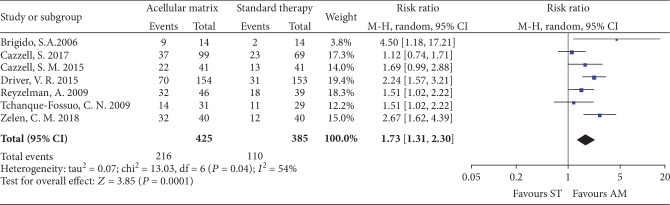
Forest plot of complete healing rate at 12 weeks.

*(1) Complete Healing Rate at 12 Weeks*. There were 7 studies [22–26, 28, 29] involving 810 patients who reported the incidence of complete healed ulcers in 12 weeks, with 425 patients randomized to receive AM therapy and 385 patients randomized to receive ST. After 12 weeks of treatments, the complete healing rate in the AM group was higher than that in the ST group (RR = 1.73, 95% CI: 1.31 to 2.30, *P* = 0.0001; Figure 3), using a random effects model (*I*^2^ = 54%).

**Figure 4:**
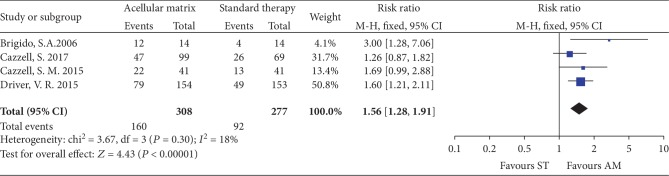
Forest plot of complete healing rate at 16 weeks.

*(2) Complete Healing Rate at 16 Weeks*. Four studies [22, 24–26] involving 585 patients reported the complete healing rate at 16 weeks, 308 patients and 277 patients were randomly assigned to the AM group and ST group, respectively. The pooled result showed that complete healing rate at 16 weeks in the AM group was also higher than that in the ST group (RR = 1.56, 95% CI: 1.28 to 1.91, *P* < 0.00001; *I*^2^ = 18%; Figure 4).

**Figure 5:**
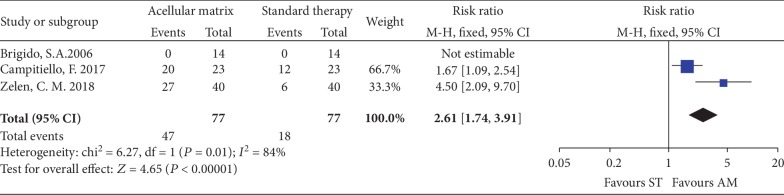
Forest plot of complete healing rate at 6 weeks.

*(3) Complete Healing Rate at 6 Weeks and 28 Weeks*. Only 3 studies [22, 27, 28] involving 154 patients reported the incidence of complete healed ulcers in 6 weeks, with 77 patients randomized to each group. No significant differences existed between the two groups (RR = 2.62, 95% CI: 0.88 to 7.84, *P* = 0.08; I^2^ = 84%; Figure 5). In one study [29] consisting of 12-week active phase and 16-week follow-up phase, there was no significant difference (*P* = 0.297) in the complete healing rate between the AM group (48.4%) and the ST group (48.3%) at 28 weeks.

#### 3.4.2. Time to Complete Heal

**Figure 6:**
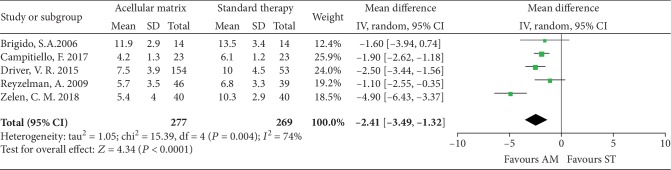
Forest plot of time to complete heal.

Five studies [22–24, 27, 28] involving 546 patients reported suitable data of complete healing time and were included in this meta-analysis. As heterogeneity test revealed statistical significance (*I*^2^ = 74%), a random effects model was used. The complete healing time in the AM group was shorter than that in the ST group (MD = −2.41; 95% CI: -3.49 to -1.32, *P* < 0.0001; Figure 6).

#### 3.4.3. Adverse Events

**Figure 7:**
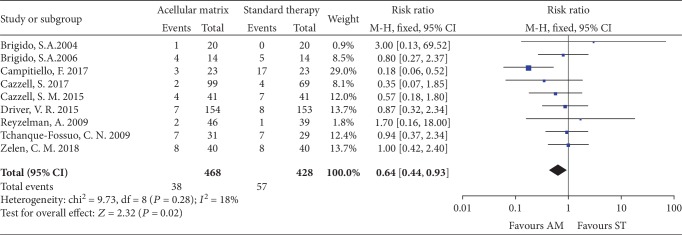
Forest plot of adverse events.

All studies reported adverse events, and only the adverse events related to AM and DFU were pooled. Most of them were diabetic foot infection, amputation, and seroma. The adverse events in the AM group were fewer than that in the ST group (RR = 0.64, 95% CI: 0.44 to 0.93, *P* = 0.02; *I*^2^ = 18%; Figure 7).

#### 3.4.4. Ulcer Depth Reduction/Ulcer Area Reduction/Quality of Life

One study [21] reported that there was a significant (*P* < 0.001) difference between the AM group (89.1%) and the ST group (25%). Two studies [21, 28] reported the mean reduction in ulcer area which ranged from 62% to 73.1% in the AM group and 34.2% to 52% in the ST group. Quality of life was evaluated in two studies. One study [26] involving 168 patients reported that there were no significant differences between the two groups for the total source or any of the eight areas, assessed by the SF-36 v2.0 (Optum, Inc.). However, another [24] showed significant improvements in physical functioning and bodily pain for the AM group, using the same evaluation scale.

### 3.5. Sensitivity Analysis

**Figure 8:**
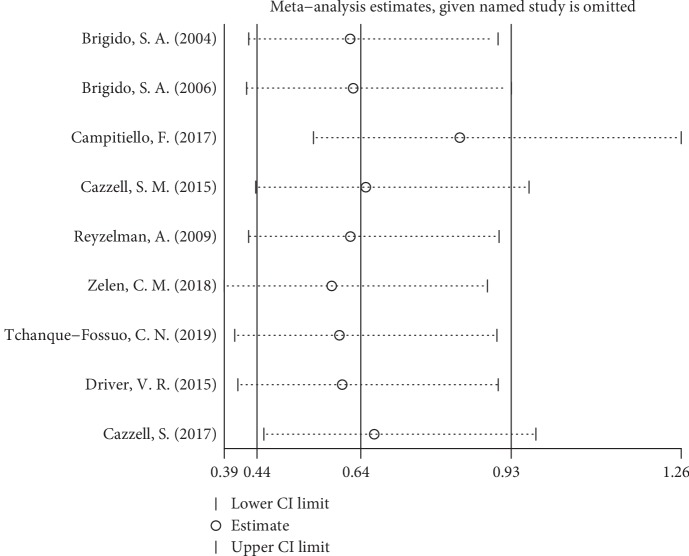
Sensitivity analysis of adverse events.

We performed sensitivity analysis by omitting one study in each turn and re-estimating the outcome. Sensitivity analysis did not identify any marked difference in the relative risk and mean difference with respect to complete healing rate at 12 weeks and 16 weeks, and time to complete heal, indicating good reliability of the outcomes. The pooled results of adverse events were as follows: RR = 0.64, 95% CI: 0.44 to 0.93, *P* = 0.02; *I*^2^ = 18%. However, as shown in Figure 8, the results changed when the study of Campitiello et al. [27] was removed: RR = 0.84, 95% CI: 0.55 to 1.26, *P* = 0.39; *I*^2^ = 0%.

### 3.6. Publication Bias

The publication bias was assessed by Egger's test and Begg's test. As shown in Table 2, the *P* values of Egger's test and Begg's test were all greater than 0.05 for ulcer complete healing rate at 12 weeks, time to heal, and adverse events, indicating no significant evidence of publication bias existed.

## 4. Discussion

In the present study, we performed a meta-analysis of 9 RCTs involving 897 patients and evaluated the efficacy and safety of AM therapy for DFUs. It was found that AM therapy was significantly associated with a higher complete healing rate at 12 weeks and 16 weeks, a shorter complete healing time, and fewer adverse events.

To our knowledge, this is the first meta-analysis to evaluate the effectiveness and safety profile of AM for patients with DFUs. Reyzelman et al. [10] performed a quantitative analysis of 3 RCTs to estimate the effectiveness of one specific human acellular dermal matrix (ADM; Graftjacket regenerative tissue matrix) in healing DFUs. Xue et al. [30] conducted a meta-analysis of 5 RCTs and assessed the efficacy and safety of allogenic ADM for DFUs. A recent meta-analysis of 6 RCTs [31] suggested that compared with the merely ST, patients in ADM group had a higher complete healing rate and faster time to heal. Moreover, no significant difference existed in adverse events between both groups. Different from our study, the studies mentioned above only involved one or more different varieties of ADM, which are a part of AMs. As we know, the AMs are derived not only from human and animal skin, called ADM, but also from other tissues, such as porcine small intestinal submucosa, urinary bladder matrix, and pericardium [13, 14]. The second difference is that two RCTs [16, 17] included in Guo's study were ongoing and only partial results were analyzed, whereas we incorporated the complete results of these 2 RCTs [26, 28]. The larger sample size probably enhanced the power of analysis.

In our meta-analysis, the results revealed that AM therapy could promote the healing of DFUs. The likelihood of ulcer complete healing in AM group is 1.73 and 1.56 times more than the ST group at 12 weeks and 16 weeks, respectively. On the basic of ST, AM therapy could further shorten the complete healing time for patients with DFUs (MD = −2.41; 95% CI: -3.49 to -1.32). These finding are robust as sensitivity analysis had confirmed that omitting any study would not change the direction of the outcomes. Considering that the estimation of the complete healing time is largely based on the trend of complete healing rate and thus influenced by the different durations of follow-up, we also analyzed this outcome separately according to the follow-up duration. The pooled results still supported the above conclusion. For the outcome of complete healing rate at 6 weeks, it was shown that between the two groups, no significant difference existed. Here, only 2 studies [27, 28] were analyzed while another [22] reported that no ulcers completely healed in 6 weeks. The heterogeneity was high, and it might result from the different severities of ulcers and different AM products. Therefore, we should look at this result with caution. It may indicate that it is difficult to show a great superiority of AM therapy over ST in a relatively short period of time. After all, DFUs usually take a long time to be cured. Several studies have shown that the mean or median healing time ranged from 2 to 4 months, even for those treated with AMs [32–35]. Further studies are needed to explore the short-term effects of AMs.

In the wound care community, concerns have been expressed that AM therapy might lead to more adverse events, such as infection and graft rejection [13, 36]. Our meta-analysis shows that people in the AM group have fewer adverse events compared to those in ST group (RR = 0.64, 95% CI: 0.44 to 0.93, *P* = 0.02). To be worthy of attention, only 1 of 9 studies reported a significant difference of adverse events between the two groups. When the study [27] was removed, the heterogeneity decreased from 18% to 0%, and the pooled result changed: RR = 0.84, 95% CI: 0.55 to 1.26, *P* = 0.39. This may be explained by the different severities of ulcers. In the study of Campitiello et al., the ulcers with grade 3 Wagner classification were more serious than those in other studies. However, the study was not dropped from the meta-analysis for the following reasons: (1) it satisfied all the inclusion criteria and was not related to the exclusion criteria; (2) reserving this study could bring our meta-analysis closer to reality, considering that there are a considerable part of DFUs classified as Wagner grade 3 in clinical practice. In another view, our meta-analysis at least proves that AM therapy would not increase the incidence of adverse events, indicating a safety profile.

Endpoints such as ulcer area reduction and ulcer depth reduction are the clinically relevant outcomes reflecting the effectiveness of AM therapy. Although some studies [21, 26, 28] reported that participants in the AM group had more reduction in the ulcer area or depth than those in the ST group, we could not conduct a meta-analysis due to the lack of suitable data. Other endpoints such as quality of life and cost-effectiveness also need to be considered. In this meta-analysis, only 2 RCTs [24, 26] evaluated the quality of life and their conclusions were not consistent. Additionally, while AM therapy leads to higher economic costs, the potential savings associated with accelerated closure of these chronic, refractory DFUs should be considered. It was disappointing that no RCTs compare the cost-effectiveness of AM therapy with ST. Researchers need to pay attention to these outcomes in their further studies.

In our meta-analysis, the quality of included clinical trials is middle to excellent and the strength of evidence is moderate. However, a few limitations should be acknowledged. First, although a comprehensive literature search was conducted and Begg's test or Egger's test revealed no publication bias existed, some negative outcomes might not be published because these studies were always related to commercial corporations. And only 2 studies [25, 29] assessing the same AM product derived from other tissues rather than from the skin were included. Second, the differences in AM products and standard therapies in each study may have affected our outcomes. Third, although there were 9 RCTs included, the sample size of each study was relatively small. For some outcomes, the limited number of studies were included and analyzed. Fourth, the criterion of adverse events was not very clear in some of the trials. Fifth, the included patients usually had adequate perfusion and a great majority of foot ulcers was full thickness and noninfective; thus, our findings may not be applicable to all patients with DFUs. Finally, due to the short-term follow-up duration of all studies, we could not explore the long-term effects of AM therapy.

## 5. Conclusion

In conclusion, the present meta-analysis suggests that AM therapy as an adjuvant treatment could further promote the healing of full-thickness, noninfected, and nonischemic diabetic foot ulcers. AM therapy also has a safety profile. However, because of various limitations, more large well-designed randomized clinical trials with long follow-up duration are needed to further explore the efficacy and safety of AM therapy for DFUs.

## Figures and Tables

**Table:** (a) Entry criteria, intervention and control groups, and primary outcome for the included RCTs

Study	Main entry criteria	Intervention treatment	Control treatment	Additional treatments in both groups	Primary outcome	Follow-up, weeks
Brigido et al. [21]	DFU: Full thickness, > 1 cm^2^in size, present for ≥6 weeks without epidermal coverage, on the leg or foot	Received a single application of the GJ-ADM	ST: Curasol wound gel, gauze dressings	Offloading, debridement	NR	4
Brigido et al. [22]	T1/T2DM; DFU: Full thickness, chronic (present for ≥6 weeks without epidermal coverage), absence of active infection	Received a single application of the GJ-ADM	ST: Wound gel	Sharp debridement, dressing, offloading	NR	16
Reyzelman et al. [23]	T1/T2DM; ≥18 years of age; adequate circulation to the affected extremity; DFU: UT grade 1 or 2; 1-25 cm^2^ in size, absence of infection	Received a single application of the GJ-ADM	ST: Moist-wound therapy (alginates, foams, hydrogels or hydrocolloids)	Debridement, dressing, offloading, systemic antibiotic treatment	Proportion of ulcers that completely healed at 12 weeks	12
Driver et al. [24]	T1/T2DM; ≥18 years of age; HbA1c<12%; adequate vascular perfusion; DFU: Neuropathic, Wagner grade 1 or 2, 1 cm^2^ ≤ area ≤ 12 cm^2^, depth ≤ 5 mm, distal to the malleolus, present for ≥30 days	Received single or multiple applications of IFRT	ST: Moist wound therapy (sodium chloride gel, nonadherent foam dressing, outer gauze wrap)	Debridement, dressing, offloading	Percentage of subjects with complete closure of ulcer as assessed by the investigator	16
Cazzell et al. [25]	T1/T2DM; ≥18 years of age; HbA1c ≤ 12%; adequate arterial blood flow; DFU: Neuropathic, Wagner grade 1-2, 0.5 cm^2^ to 10 cm^2^ in size, plantar surface of the foot, a duration ≥6 weeks but<12 months; absence of infection	Received weekly applications of OASIS® ultra tri-layer matrix	ST: Dressing, debridement	Offloading	The proportion of Subjects with complete ulcer closure over the 12-week treatment period	16
Cazzell et al. [26]	21-80 years of age; adequate circulation to the affected area; DFU: Wagner grades 1-2, 1 cm^2^ ≤ area < 25 cm^2^, absence of infection	Received one or two applications of D-ADM or GJ-ADM	ST: moist wound treatment (alginate, foam, or hydrogel dressings)	Debridement, offloading, dressing	The proportion of chronic DFUs completely closed at the end of 12 weeks	24
Campitiello et al. [27]	Diabetes; >18 years of age; ABI ≥ 0.5; DFU: Wagner grade 3	Treated with IFWN	ST: wet dressing	Offloading, antibiotics, compression therapy	Percentage of patients with complete closure	6
Zelen et al. [28]	T1/T2DM; ≥18 years of age; HbA1c < 12%; adequate circulation to the affected extremity; DFU: >1 cm^2^ in size, on the foot, present for ≥4 weeks, absence of infection	Received weekly applications of HR-ADM	ST	Dressing, debridement, offloading, systemic antibiotics	The difference between the 2 groups in the proportion of ulcers healed at 6 weeks	12
Tchanque-Fossuo et al. [29]	T1/T2DM; 18-85 years of age; HbA1c ≤ 12%; 0.8 ≤ ABI ≤ 1.4 or toe − arm index ≥ 0.6; DFU: full thickness (not extending to the bone, muscle, or tendon), 0.5 cm^2^ ≤ area ≤ 25 cm^2^, present for ≥4 weeks, absence of infection	Treated with oasis matrix	ST	Nonadherent gauze dressing, Iodosorb gel, offloading	The percentage of patients who achieved complete ulcer closure by 12 weeks of treatment	28

**Table:** (b) Participants' descriptive demographics and wound characteristics for the included RCTs

Study	Group	Sample size	Age (years)	Male (%)	ABI	BMI (kg/m^2^)	HbA1c (%)	Ulcer grade (Wagner or UT)	Ulcer area (cm^2^)	Ulcer duration (weeks)
Brigido S. A. [22]	GJ-ADM	20	NR	NR	NR	NR	NR	NR (full thickness)	9.7	25
	ST	20	NR	NR	NR	NR	NR	NR (full thickness)	5.4	27
Brigido S. A. [22]	GJ-ADM	14	61.4 (4.2)	NR	NR	NR	8.1 (1.0)	Wagner grade 2	NR	NR
	ST	14	66.2 (4.4)	NR	NR	NR	7.9 (0.6)	Wagner grade 2	NR	NR
Reyzelman A. [23]	GJ-ADM	47	55.4 (9.6)	NR	NR	33.1 (6.7)	8.2 (2.0)	UT grades 1A-2A	3.6 (4.3)	23.3 (22.4)
	ST	39	58.9 (11.6)	NR	NR	34.6 (8.5)	8.0 (1.6)	UT grades 1A-2A	5.1 (4.8)	22.9 (29.8)
Driver V. R. [24]	IDRT	154	55.8 (10.6)	76.6	NR	34.0 (7.2)	8.0 (1.8)	Wagner grade 1 or 2	3.5 (2.5)	44.0 (70.1)
	ST	153	57.3 (9.8)	74.5	NR	34.1 (8.4)	8.2 (1.9)	Wagner grade 1 or 2	3.7 (2.7)	43.3 (59.7)
Cazzell S. M. [25]	OASIS	41	57.1 (10.9)	78	NR	NR	NR	Wagner grade 1 or 2	2.1 (2.3)	21.3 (12.3)
	ST	41	56.6 (10.8)	73	NR	NR	NR	Wagner grade 1 or 2	2.6 (7.5)	22.2 (13.5)
Cazzell S. [26]	D-ADM	71	59.1 (12.8)	80.3	NR	32.6 (8.3)	8.5 (1.8)	Wagner grade 1 or 2	3.9 (4.2)	40.0 (71.6)
	GJ-ADM	28	58.5 (9.8)	71.4	NR	31.4 (5.1)	7.6 (1.4)	Wagner grade 1 or 2	3.3 (2.7)	36.8 (53.6)
	ST	69	56.9 (10.9)	73.9	NR	32.8 (6.9)	8.4 (1.9)	Wagner grade 1 or 2	3.6 (3.6)	36.4 (38.8)
Campitiello F. [27]	IFWM	23	64.0 (8.9)	65.2	0.92 (0.1)	28.5 (2.5)	7.9 (0.8)	Wagner grade 3	NR	38.8 (12.6)
	ST	23	62.1 (7.7)	56.5	0.94 (0.1)	28.9 (2.7)	7.8 (0.8)	Wagner grade 3	NR	39.5 (9.9)
Zelen C. M. [28]	HR-ADM	40	59.0 (12.0)	70	NR	35.0 (7.9)	7.8 (1.5)	UT grades 1-2	3.2 (4.0)	NR
	ST	40	62.0 (13.0)	60	NR	34.0 (8.8)	7.6 (1.4)	UT grades 1-2	2.7 (2.4)	NR
Tchanque-Fossuo C. N. [29]	OASIS	31	61.9 (8.6)	94.7	1.10 (0.1)	36.5 (11.6)	7.7 (1.6)	NR (full thickness)	3.1 (3.8)	10.9 (7.6)
	ST	29	63.3 (9.1)	89.5	1.07 (0.1)	36.5 (6.6)	8.6 (1.7)	NR (full thickness)	1.3 (0.9)	21.7 (36.0)

Continuous data are presented in mean (standard difference). NR: not reported; DFU: diabetic foot ulcer; DM: diabetic mellitus; UT: University of Texas; HbA1c: glycosylated hemoglobin; ABI: ankle brachial index; BMI: body mass index; ST: standard therapy; ADM: acellular dermal matrix; GJ-ADM: GraftJacket ADM; D-ADM: DermACELL ADM; IDRT: Integra Dermal Regeneration Template; IFWM: Integra Flowable Wound Matrix; HR-ADM: human reticular ADM; CG-ADM: CGBio ADM.

**Table 2:** Egger's test and Begg's test for the outcomes.

Outcome	P	
	Egger's test	Begg's test
Complete healing rate at 12 weeks	0.617	1.000
Time to complete heal	0.438	0.806
Adverse events	0.766	0.754

## Data Availability

The data supporting this meta-analysis are from previously reported studies, which have been cited. The processed data are available from the corresponding author upon request.
